# AaABCG40 Enhances Artemisinin Content and Modulates Drought Tolerance in *Artemisia annua*

**DOI:** 10.3389/fpls.2020.00950

**Published:** 2020-06-26

**Authors:** Xueqing Fu, Hang Liu, Danial Hassani, Bowen Peng, Xin Yan, Yuting Wang, Chen Wang, Ling Li, Pin Liu, Qifang Pan, Jingya Zhao, Hongmei Qian, Xiaofen Sun, Kexuan Tang

**Affiliations:** Joint International Research Laboratory of Metabolic & Developmental Sciences, Key Laboratory of Urban Agriculture (South) Ministry of Agriculture, Plant Biotechnology Research Center, Fudan-SJTU-Nottingham Plant Biotechnology R&D Center, Shanghai Jiao Tong University, Shanghai, China

**Keywords:** *Artemisia annua*, artemisinin, pleiotropic drug resistance (PDR) transporter, drought tolerance, abscisic acid

## Abstract

The phytohormone Abscisic acid (ABA) regulates plant growth, development, and responses to abiotic stresses, including senescence, seed germination, cold stress and drought. Several kinds of researches indicate that exogenous ABA can enhance artemisinin content in *A. annua*. Some transcription factors related to ABA signaling are identified to increase artemisinin accumulation through activating the artemisinin synthase genes. However, no prior study on ABA transporter has been performed in *A. annua*. Here, we identified a pleiotropic drug resistance (PDR) transporter gene *AaPDR4/AaABCG40* from *A. annua*. *AaABCG40* was expressed mainly in roots, leaves, buds, and trichomes. GUS activity is primarily observed in roots and the vascular tissues of young leaves in *proAaABCG40*: *GUS* transgenic *A. annua* plants. When *AaABCG40* was transferred into yeast AD12345678, yeasts expressing *AaABCG40* accumulated more ABA than the control. The *AaABCG40* overexpressing plants showed higher artemisinin content and stronger drought tolerance. Besides, the expression of *CYP71AV1* in OE-*AaABCG40* plants showed more sensitivity to exogenous ABA than that in both wild-type and i*AaABCG40* plants. According to these results, they strongly suggest that AaABCG40 is involved in ABA transport in *A. annua*.

## Introduction

Artemisinin, isolated from the traditional Chinese medicine *A. annua*, is extensively used for the treatment of malaria (Weathers et al., 2006). Artemisinin Combination Therapies (ACTs) are presently recommended by WHO (World Health Organization) as the preferred drug to fight the malaria (World Health Organization, 2017). Considerable effort has been expended to determine the artemisinin biosynthetic pathway (**Figure S1**). The mevalonate (MVA) pathway and the methylerythritol phosphate (MEP) pathway produce the precursors isopentenyl diphosphate (IPP) and its isomer dimethylallyl diphosphate (DMAPP) (Vranová et al., 2013). Farnesyl diphosphate synthase (FPS) catalyzes IPP and DMAPP to synthesize farnesyl diphosphate (FPP) (Schramek et al., 2010). After that, amorpha-4, 11-diene synthase (ADS) catalyzes the cyclization reaction using FPP as the substrate to synthesize amorpha-4, 11-diene (Bouwmeester et al., 1999; Mercke et al., 2000). Then amorpha-4, 11-diene is oxidized to artemisinic alcohol, and further catalyzed into artemisinic aldehyde by the cytochrome P450 monooxygenase (CYP71AV1) (Ro et al., 2006; Teoh et al., 2006). Artemisinic aldehyde D11 (13) reductase (DBR2) catalyzes artemisinic aldehyde to form dihydroartemisinic aldehyde (Zhang et al., 2008). Then dihydroartemisinic aldehyde is converted into the direct precursor of artemisinin, dihydroartemisinic acid (DHAA), catalyzed by aldehyde dehydrogenase (ALDH1) (Teoh et al., 2009). Subsequently, artemisinin is synthesized *via* a nonenzymatic reaction (Brown and Sy, 2004). Alternatively, CYP71AV1 and ALDH1 catalyze the artemisinic aldehyde to form artemisinic acid (Ro et al., 2006; Teoh et al., 2009). Artemisinic acid synthesized arteannuin B *via* a nonenzymatic photo-oxidized reaction (Brown and Sy, 2007). In addition, artemisinin biosynthesis occurs in the glandular trichomes of *A. annua*, containing two stalk, two basal, and three pairs of secretory cells (Duke and Paul, 1993; Olsson et al., 2009).

The limited supply of artemisinin which is due to its low content (0.1%-1.0% dry weight) in *A. annua* has urged its production improvement through developing a new kind of *A. annua* plant with higher content of artemisinin (Tang et al., 2014). It is well-known that the artemisinin content is enhanced by the treatment of exogenous ABA (Abscisic acid) in *A. annua* (Jing et al., 2009). The phytohormone ABA is a phytohormone with the sesquiterpene structure, that plays important roles in several biological processes, such as senescence, seed germination, and root elongation, as well as responses to cold stress, drought and salt (Finkelstein et al., 2002; Zhu, 2002; De Smet et al., 2006; Bi et al., 2017; Sun et al., 2018). More studies showed that ABA was mainly synthesized in leaves (Hartung et al., 2002). McAdam et al. propose that the decline in leaf water status causes ABA biosynthesis, that regulates the stomatal closure. Then ABA is transported from the leaves to the roots to promote root growth (McAdam et al., 2016a; McAdam et al., 2016b). The biosynthesis of ABA was also autonomously occurred in guard cells and triggered stomatal closure (Endo et al., 2008; Bauer et al., 2013). In the past several decades, research on ABA has focused on the mechanism of ABA regulating artemisinin biosynthesis in *A. annua* (Zhang et al., 2013; Zhang et al., 2015; Zhong et al., 2018). Several studies indicated that ABA transporter is crucial for the ABA function (Taylor et al., 2000; Boursiac et al., 2013; Zhang et al., 2014). However, the molecular basis of ABA transport is currently unknown in *A. annua*.

Several ABA transporters in plants have been recently reported, some of which belong to ATP-binding-cassette (ABC) transporter family. ABC transporters are one of the biggest protein families in plants, which act as ATP-driven transporters for a wide range of substrates, including terpenoids, lipids, vitamins, organic acids, and ions (Theodoulou, 2000; Lee et al., 2005; Sugiyama et al., 2006; Kang et al., 2010; Fu et al., 2017). In plants, ABC transporters are divided into eight subfamilies (Verrier et al., 2008). In particular, the pleiotropic drug resistance (PDR) transporters are the essential branch of the ABCG subfamily (Rea, 2007). In *Arabidopsis*, AtPDR12/AtABCG40, a member of PDR subfamily of ABC transporters, mediated cellular ABA uptake and involved in the detoxification of Pb^2+^ (Lee et al., 2005; Kang et al., 2010). Subsequently, *AtABCG25* was isolated from *Arabidopsis* and encoded an ABCG subfamily transporter. These results suggested that AtABCG25 functioned as an exporter of ABA and also controlled the intercellular ABA signaling in *Arabidopsis* (Kuromori et al., 2010). AtABCG22, an ABCG transporter closely related to AtABCG25, was identified to be associated with stomatal regulation in *Arabidopsis* and considered as a candidate ABA transporter, the functions of which have not been demonstrated in the ABA signaling and biosynthesis pathways (Kuromori et al., 2011). In the process of ABA signaling, AtABCG25 acts as a mediator in exporting ABA from vascular tissues, while AtPDR12/AtABCG40 plays a role in importing ABA into guard cells (Kang et al., 2010; Kuromori et al., 2010). Simultaneously, *AtDTX50* encoded a Multidrug and Toxic Compound Extrusion (MATE) protein, which was identified and found to be expressed in both guard cells and vascular tissues of *Arabidopsis thaliana*. When *AtDTX50* was expressed in both *Escherichia coli* and *Xenopus oocyte*, it functioned as an ABA efflux transporter (Zhang et al., 2014). In addition, the function of an NRT1.2 in the nitrate transporter (AIT1) as a regulator is to control the ABA pool size at the primary site of ABA synthesis (Kanno et al., 2012). Here, we report that a PDR transporter AaPDR4/AaABCG40 was cloned from *A. annua*. AaABCG40 was involved in ABA transport. Overexpressing *AaABCG40* could enhance artemisinin content and drought tolerance.

## Experimental Procedures

### Plant Materials

*A. annua* seeds (Huhao 1) obtained from Chongqing province, were developed by our group in Shanghai. Plants were grown under a 16/8 h light/dark photoperiod at 25°C in the greenhouse. Tobacco (*Nicotiana benthamiana*) was grown under the same conditions as *A. annua* (Shen et al., 2016).

### Isolation and Characterization of *AaABCG40*

ABC transporter proteins were identified by using the HMM model (PF00005.27) from Pfam (http://pfam.xfam.org/) for searching against *A. annua* protein databases and reduced sequence redundancy by CD-HIT (Shen et al., 2018). *A. annua* ABC transporters were analyzed using the Conserved Domain Database (CDD) (Çakır and Kılıçkaya, 2013). The phylogenetic tree analysis was performed using MEGA7 *via* the neighbor-joining method, and the bootstrap analysis was performed using 1000 replicates (Kumar et al., 2016). The ABC transporter protein sequences from *A. annua* were aligned with ClustalX. The Heatmap was generated using the MultiExperiment Viewer (MeV). The full-length of *AaABCG40* sequence was predicted from the *A. annua* genome database. 500 ng total RNA isolated from the leaves of *A. annua* was used to synthesize cDNA, and the full-length of *AaABCG40* was amplified using the specific primers (**Table S1**).

### Real-Time Quantitative PCR

To check the expression level of the putative genes, total RNA was extracted using the RNeasy Kit (Qiagen, Germany). Fresh leaves, roots, and aerial tissues of 5-month-old *A. annua* were collected at various developmental stages and grounded to powder in liquid nitrogen with mortar and pestle (Fu et al., 2017). Before cDNA synthesis, DNase (DNase I Kit, Takara, Japan) treatment was applied to digest the genomic DNA. Subsequently, cDNA was reverse transcribed using a reverse transcription kit (Promega, USA). RT-qPCR was carried out using the Roche Lightcycler ^®^ 96 (Roche, Mannheim, Germany) with Fast Start Universal SYBR Green Master Mix (Roche Diagnostics, Germany) as described previously (He et al., 2017). qRT-PCR was performed in three independent experimental replicates. Calculation of the relative expression level was performed using the 2^-ΔΔct^ method (Livak and Schmittgen, 2001). **Table S1** summarizes the primers.

### Construction and Transformation of *A. annua*

To construct the RNAi lines, the 300 bp non-conservative domain coding sequence of *AaABCG40* cDNA was cloned in pENTR gateway cloning vector and further inserted into pHELLSGATE12 *via* LR recombination reaction (Invitrogen, Carlsbad, CA, USA). Alternatively, the *AaABCG40* open reading frame was inserted into pHB-GFP overexpression vector. Both overexpressed and knocked down vectors were transformed into *A. annua* using *Agrobacterium*-mediated transformation (*Agrobacterium tumefaciens* strain EHA105). Empty pHB-GFP and pHELLSGATE12 were used as negative controls. After 3-4 months the transgenic lines were shifted to pots and transferred to the greenhouse.

### Subcellular Localization of AaABCG40

The recombinant plasmid (pHB-*AaABCG40*-*GFP*) was transferred into *A. tumefaciens* strain GV3101 for *Nicotiana benthamiana* leaves transient expression (Voinnet et al., 2003). The fusion protein AaABCG40-GFP and PIP1-mCherry protein locate at the plasma membrane were injected into tobacco leaf together to confirm the localization of AaABCG40 (Siefritz et al., 2002). After 2-3 days, the GFP fluorescence could be observed using Leica TCS SP5-II confocal laser microscopy (Leica, Wetzlar, Germany).

### Molecular Cloning of *AaABCG40* Promoter and Promoter-GUS Fusions in Transgenic *A. annua*

The promoter of *AaABCG40* was predicted from *A. annua* genomic databases (Shen et al., 2018). The promoter region of *AaABCG40* was amplified with *AaABCG40*-specific primers using the genomic DNA of the *A. annua* leaves as the template (**Table S1**). The promoter region was amplified containing *Pst*I and *BamH*I restriction sites and inserted into pCAMBIA1391Z vector. Subsequently, the recombinant plasmid (pCAMBIA1391Z-proAaABCG40-GUS) was transferred into *A. tumefaciens* strain EHA105 for the plant transformation. All the primers mentioned in this experiment are listed in **Table S1**. Histochemical staining for GUS activity in transgenic plants was performed according to previous protocol (Jefferson, 1987).

### Artemisinin Content Analysis by HPLC-ELSD

To measure the artemisinin content of both overexpressed and RNAi line, fresh leaves were collected and stored at 45°C for 48 h, dried leaves were powdered, and 0.1 g/sample was extracted twice with 1 ml methanol and disrupted by an ultrasonic processor (Shanghai Zhisun Instrument Co. Ltd model JYD-650) at 40°C and 55 Hz for 30 min. Centrifuging at 12,000 rpm for 10 min, the supernatant was collected and moved to a new 2 ml tube. The above steps were carried out one more time to maximize the total extraction. The samples were then passed through a nitrocellulose 0.25 μm pore size Sartorious ^®^ membrane. The samples were then injected into a Waters Alliance 2695 HPLC system coupled with a Waters 2420 ELSD detector (Milford, MA, USA) using pure artemisinin as standard (sigma). The HPLC condition was as described previously (Chen et al., 2012). Three biological repeats were applied for each sample.

### Measurement of ABA Concentration

The ABA concentration was measured using a Phytodetek ABA enzyme immunoassay test kit (Elisa, Agdia, Elkhart, USA). Fresh leaves were ground into powder in liquid nitrogen. Then 100 mg powder of each sample was extracted with 8 ml solution (80% methanol, 100 mg/L butylated hydroxytoluene, and 0.5 g/L citric acid monohydrate), and stirred overnight at 4°C in the dark. The culture was centrifuged at 12,000 rpm for 10 min at 4°C. Subsequently, the supernatant was collected in a new tube and dried. The residue was dissolved in 100 μl methanol and 900 μl of TBS buffer (50 mM Tris, 0.1 mM MgCl_2_·6H_2_O, 0.15 M NaCl, pH 7.8) and analyzed as described previously (Zhang et al., 2014).

### Functional Analysis of AaABCG40 in Yeast Cells

The CDS of *AaABCG40* was inserted into the *Spel*I and *Pst*I sites of pDR196. *AtPDR12* was cloned and inserted into the *Spel*I and *Pst*I sites of pDR196 vector as the positive control. The recombinant plasmids (pDR196-AaABCG40 and pDR196-AtPDR12) were respectively introduced into the strain AD12345678 using the lithium acetate method. The yeast transformant was incubated in 50 ml Synthetic Dextrose (SD) medium (-uracil) at 29°C with shaking at 180 rpm until OD_600_ reached at 1.0, subsequently suspended using 50 ml half-strength SD medium (-uracil) containing 50 μM ABA (Sigma-Aldrich). The cells were cultivated with shaking at 180 rpm at 29°C and collected by centrifuging at the indicated times, respectively. The cells were washed twice using the sterilized water, and followed by disrupted in methanol for 15 min at 30 Hz using acid-washed glass beads (Yu and De Luca, 2013). The supernatants were collected and filtered for ABA contents analysis. Three biological repeats were applied for each sample.

### Abscisic Acid Treatment and Drought Treatment

For hormone treatments, 100 μM ABA was used, whereas water with 1% of ethanol was used as a mock treatment. The cutting seedlings of OE-*AaABCG40* transgenic plants, i*AaABCG40* transgenic plants, and wild-type *A. annua* plants were sprayed with 100 ml ABA (100 μM), respectively, followed by sampling at 0, 1, 3, 6, 9, and 12 h for RNA extraction to analyze the gene expression. Two-month-old cutting seedlings of OE-*AaABCG40* transgenic plants, i*AaABCG40* transgenic plants, and wild-type *A. annua* plants were cultivated in pots and watered well in the growth chamber under a 16-h light/8-h dark cycle at 25°C for a week. Then the water supply was absolutely stopped. For drought treatment, water was withheld for a period of 14 days. After 14 days, the condition of all the plants was observed and recorded. The water loss was performed according to previous study (Zhang et al., 2014).

## Results

### Isolation and Characterization of *AaABCG40*

**Figure 1:**
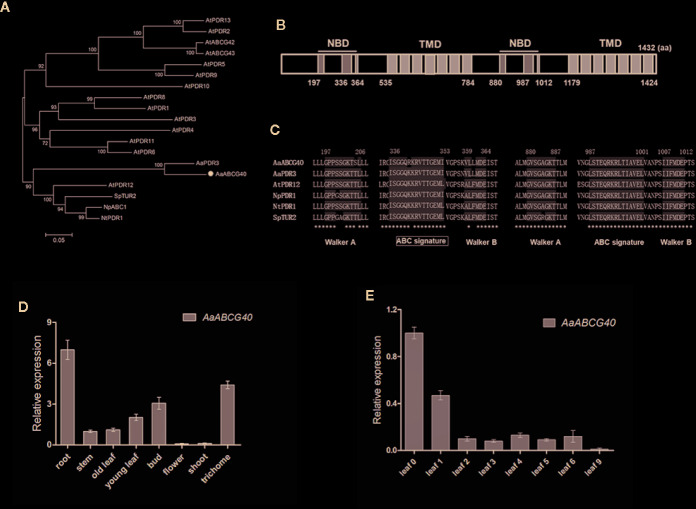
Sequence analysis of AaABCG40. **(A)** Phylogenetic analysis of AaABCG40 from *A. annua* and some known PDR transporters from *Arabidopsis*, *A. annua* AaPDR3, *N. plumbaginifolia* NpPDR1, *N. tabacum* NtPDR1, and *S. polyrrhiza* SpTUR2. The tree presented here is a neighbor-joining tree based on amino acid sequence alignment. **(B)** The structure of AaABCG40 was predicted by scanning the deduced amino acid sequence. NBD and TMD indicate the predicted location of NBDs and TMDs, respectively. **(C)** Multiple alignment of the conserved domain of known PDR transporters involved in terpene transport has the high conservation in plants. The Walker A, Walker B and ABC signature motifs are shown with shading. The identical amino acid residues in are marked by asterisks. **(D)** Relative expression of *AaABCG40* in root, stem, old leaf, young leaf, bud, flower, shoot and trichome. **(E)** Relative expression of *AaABCG40* in leaves of different developmental ages of *A. annua*. *ACTIN* was used as internal control. The error bars represent the means ± SD (standard deviation) from three biological replicates.

ABA treatment enhanced the artemisinin content through increasing the expression of artemisinin biosynthetic genes (Jing et al., 2009). In Arabidopsis, ABA transporter AtPDR12, belonging to PDR subfamily, was strongly expressed in root (Kang et al., 2010). Therefore, we want to clone and identify ABA transporter. We identified 93 ABC transporter proteins from *A. annua* by HMM research using Pfam mold (PF00005.27). Then these sequences of ABC proteins were analyzed using the Conserved Domain Database of NCBI. Identified ABC transports were aligned using ClustalW program, and the phylogenetic analysis was generated to classify them into different subfamilies. Eight PDR transporters were screened from *A. annua* (**Figure S2**). The Heatmap analysis showed that a PDR transporter gene (Aannua00284S063360) was predominately expressed in root (**Figure S3**). Therefore, this PDR transporter was further examined as the candidate transporter, that might be involved in ABA transport. The full-length cDNA of Aannua00284S063360 was cloned and assigned as *AaPDR4/AaABCG40*. *AaABCG40* is 4299 bp and encodes a protein of 1432 amino acids. The phylogenetic tree analysis with AaABCG40 and other PDR transporters, including *Arabidopsis* PDR transporters, AaPDR3, NpPDR1, NtPDR1, and SpTUR2 was performed, showing that AaABCG40 was similar to that of PDR proteins (AtPDR12, AaPDR3, NpPDR1, NtPDR1, and SpTUR2) involved in terpene transport (Van Den Brûle et al., 2002; Stukkens et al., 2005; Kang et al., 2010; Crouzet et al., 2013; Fu et al., 2017) (**Figure 1A**). AaABCG40 belongs to the full-length size PDR subfamily and contains two nucleotide-binding domains (NBD) and two transmembrane domains (TMD) (**Figure 1B**). Compared to the conserved domain of known PDR transporters involved in terpene transport, it exhibited the high conservation in plants (**Figure 1C**).

### Expression Patterns of *AaABCG40* Gene in *A. annua*

To analyze the expression pattern of *AaABCG40*, the different tissues were collected for RNA extraction from *A. annua*. RT-qPCR results showed that *AaABCG40* highly expressed in both trichomes and roots, and poorly in old leaves (**Figure 1D**). Subsequently, the *AaABCG40* expression patterns in leaves at different developmental stages were analyzed. The highest expression level in the youngest leaf (leaf 0) was observed, following a rapid reduction with the leaves aging (**Figure 1E**).

**Figure 2:**
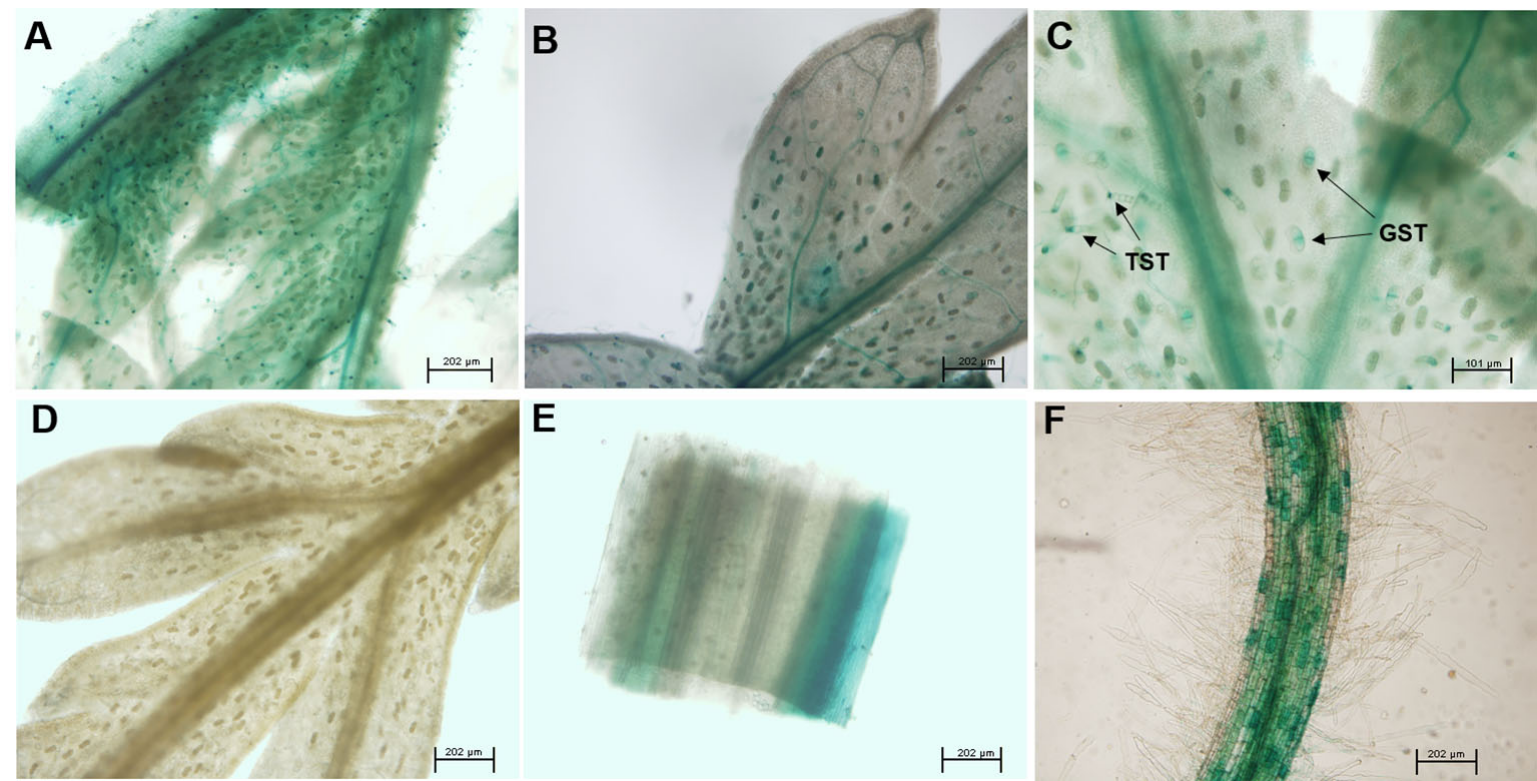
*AaABCG40* is mainly expressed in trichomes and roots. The expression of the *pro AaABCG40*-GUS was observed in **(A)** the first leaf, **(B)** the second leaf, **(C)** trichomes and vascular tissue of young leaf, **(D)** the ninth leaf, **(E)** stem and **(F)** the lateral root. GST: glandular secretory trichome; TST, T-shaped trichome.

To further analyze the expression pattern of *AaABCG40* in *A. annua,* the predicted promoter sequence from the genome database was cloned and inserted into the vector pCAMBIA1391Z carrying *GUS* reporter gene. The recombinant plasmid was further introduced into *A. annua* plants. The GUS staining was mainly active in the vascular tissues of leaves and roots in transgenic plants, following with high expression in trichomes (**Figure 2**). Similarly, GUS staining was primarily restricted to the hypocotyls, roots, and vascular veins of leaves in the *pAtABCG25-GUS* transgenic plants (Kuromori et al., 2010). It was also observed that the GUS signals of the *pAtABCG40-GUS* transgenic plants was predominantly active in roots and the leaves of young plantlets (Kang et al., 2010).

### AaABCG40 Was a Plasma Membrane-Localized Protein

**Figure 3:**
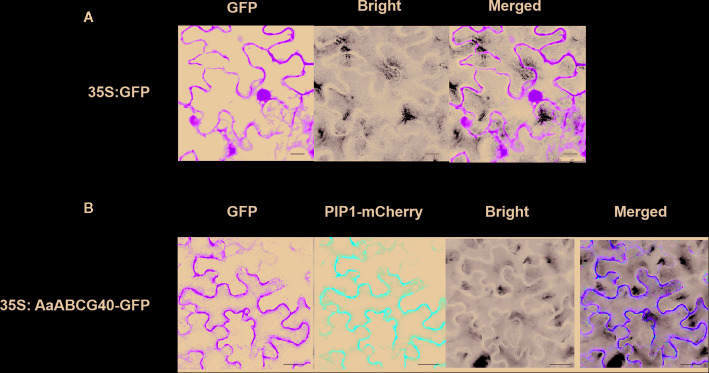
The subcellular localization of AaABCG40. **(A)** Localization of 35S: GFP in tobacco leaves. **(B)** AaABCG40 protein co-localized with plasma membrane integral protein PIP1 on the plasma membrane of tobacco leaves determined through confocal microscopy. Bars = 40 μm.

To determine the subcellular localization of AaABCG40 protein, we performed a construct that produced the green fluorescent protein (GFP) fused to the C-terminal domain of AaABCG40 under control of the CaMV35S promoter. Subsequently, the *AaABCG40-GFP* recombinant plasmid was transiently co-expressed in tobacco leaves together with the reported plasma membrane marker PIP1 (Siefritz et al., 2002). Subcellular localization of the AaABCG40-GFP fusion protein was observed in plasma membrane with PIP1-mCherry (**Figure 3**). The results showed that AaABCG40 was a plasma membrane-localized protein, implying that AaABCG40 functioned as a transport through the cellular membrane.

### Overexpression of *AaABCG40* Increases Artemisinin Biosynthesis

**Figure 4:**
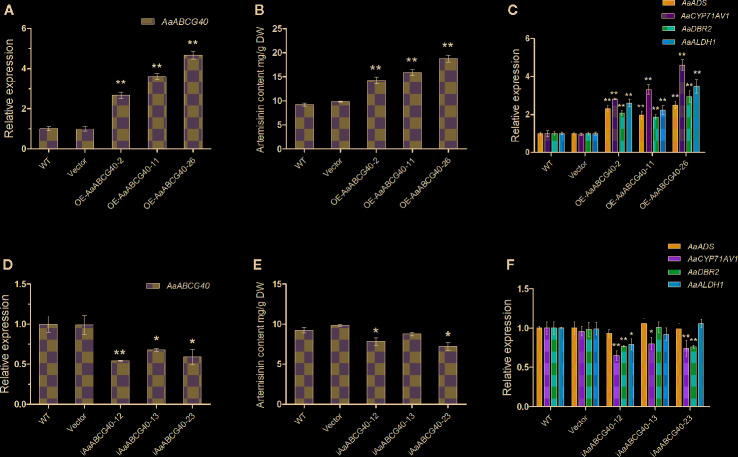
Comparative analyses of *AaABCG40* gene expression and artemisinin analyses in wild type (WT), plants transformed with the empty vector (EV), *AaABCG40*-overexpression and *AaABCG40*-RNAi plants. **(A)** Relative expression of *AaABCG40* in WT, EV and *AaABCG40*-overexpression transgenic *A. annua* lines. **(B)** The contents of artemisinin in WT, EV, and *AaABCG40*-overexpression transgenic *A. annua* lines. **(C)** Relative expression of *AaADS*, *AaCYP71AV1*, *AaDBR2,* and *AaALDH1* in WT, EV and *AaABCG40*-overexpression transgenic *A. annua* lines. **(D)** Relative expression of *AaABCG40* in WT, EV, and *AaABCG40*-RNAi transgenic *A. annua* lines. **(E)** The contents of artemisinin in WT, EV, and *AaABCG40*-RNAi transgenic *A. annua* lines. **(F)** Relative expression of *AaADS*, *AaCYP71AV1*, *AaDBR2,* and *AaALDH1* in WT, EV, and *AaABCG40*-RNAi transgenic *A. annua* lines. All data represent the means ± SD of three replicates. **P < 0.0 5, *P < 0.01, student’s *t*-test.

To further explore the function of AaABCG40, 35S::*AaABCG40* transgenic *A. annua* lines were generated. In the *AaABCG40*-overexpressing transgenic plants, the transcript levels of *AaABCG40* were markedly increased to 2.6-4.7 folds compared with the WT (**Figure 4A**). Therefore, we selected three independent lines for further analysis. The artemisinin content was measured from three independent transgenic plants by HPLC. According to our data, 35S:: *AaABCG40* transgenic *A. annua* lines tested produced about 1.54-2.03-fold artemisinin content than the control (**Figure 4B**). RT-qPCR results showed that the expression of the artemisinin biosynthetic enzyme genes *ADS*, *CYP71AV1*, *DBR2,* and *ALDH1* was increased to 2.3-2.5-, 2.8-4.6-, 1.9-2.9-, and 2.2-3.5-fold in *OE-AaABCG40*-2, 11, 26 transgenic plants, respectively (**Figure 4C**).

To further analyze the function of AaABCG40, we downregulated the *AaABCG40* expression in *A. annua*. Investigation of *AaABCG40* transcript levels by RT-qPCR showed that the *AaABCG40* expression was significantly decreased in *AaABCG40*-RNAi lines. Three independent transgenic lines (i*AaABCG40*-12, 13, 23) exhibiting a 54%-68% reduction of *AaABCG40* transcript levels were chosen for the further experiments (**Figure 4D**). In order to analyze whether other ABCG genes are affected or not, the expression levels of ABCG transporter genes from *A. annua*, which have high homology with *AaABCG40*, were analyzed by qRT-PCR. These results showed that the expression of ABCG subfamily genes of the transgenic plants had no significant difference with those of both wild type and empty vector plants (**Figure S4**). The content of artemisinin was slightly decreased, and the lowest artemisinin content was merely decreased by 17.4% of the control (**Figure 4E**). RT-qPCR results showed the transcript levels of *CYP71AV1* and *DBR2* were generally reduced to 44%-80% and 76%-77% of the control, while the transcript levels of *ADS* and *ALDH1* were not significantly downregulated (**Figure 4F**). Taken together, these data demonstrated that the change of the substrate content transported by AaABCG40 enhanced the artemisinin accumulation through activating the expression of the artemisinin synthase genes in *A. annua*.

### AaABCG40 Was an ABA Influx in Yeast Strain AD1-8

**Figure 5:**
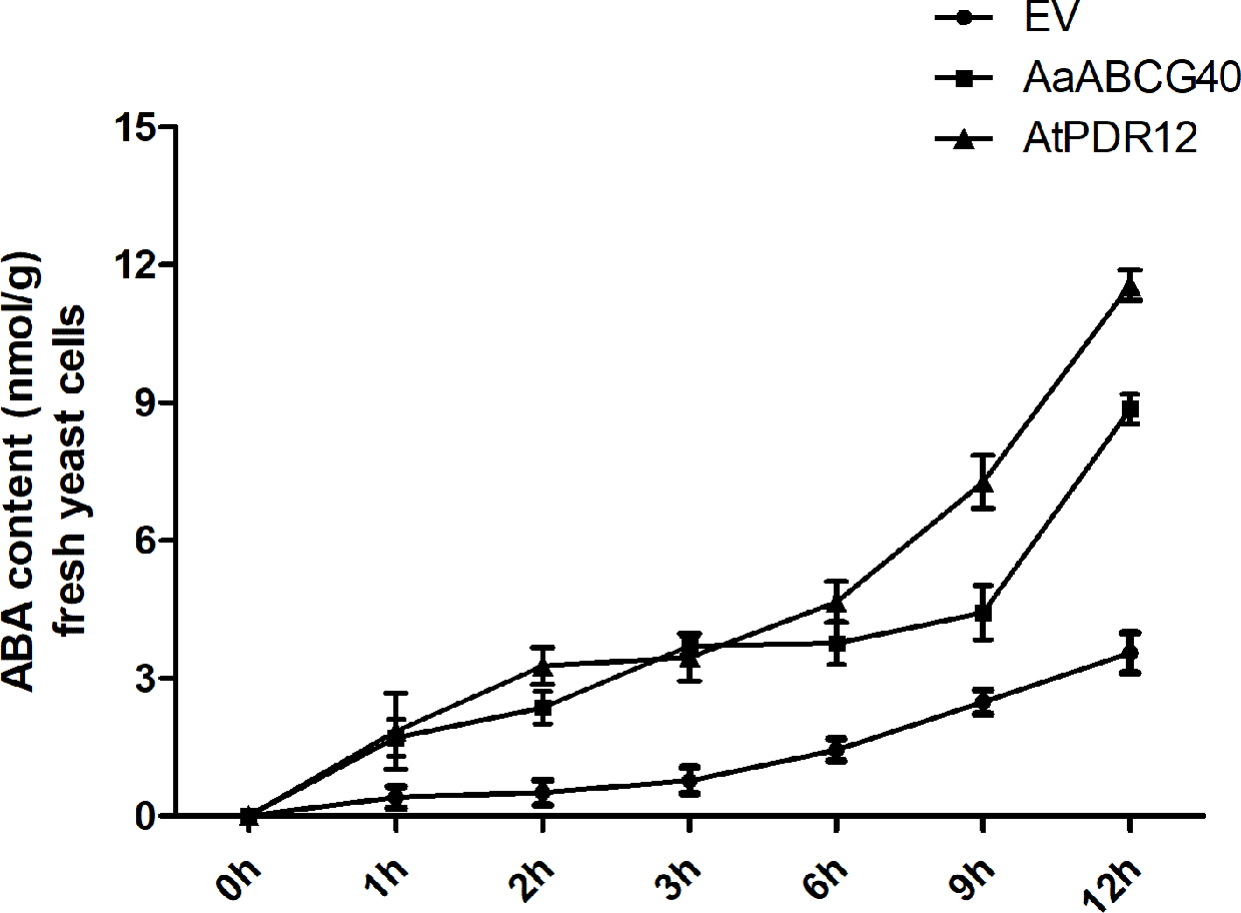
Time-dependent uptake of ABA by AD1-8 yeast cells expressing *AaABCG40* and transformed with the empty vector (EV). Yeast was incubated in half-strength SD medium containing 50 μM ABA at pH 5.9. The error bars represent the means ± SD from three biological replicates. The recombinant plasmid (pDR196-AtPDR12) was introduced into the strain AD12345678 as the positive control.

In higher plants, ABA is synthesized in leaves, and accumulated in guard cells and vascular tissues, which is then transported to other tissues (Cheng et al., 2002; Koiwai et al., 2004; Endo et al., 2008). In *Arabidopsis*, AtABCG40/AtPDR12 localized at plasma membrane was identified to function as ABA transporter (Kang et al., 2010). AaABCG40 cloned from *A. annua* had the closest evolutionary relationship to AtPDR12, and also the similar expression pattern with AtPDR12, which suggested that AaABCG40 might have a similar function in *A. annua*. Besides, ABA treatment enhanced the artemisinin accumulation through activating the expression of the synthase genes in artemisinin biosynthesis (Jing et al., 2009). Therefore, we expressed *AaABCG40* cDNA in a heterologous system, the yeast mutant strain AD12345678 (Decottignies et al., 1998). The recombinant plasmid (pDR196-AtPDR12) was introduced into the strain AD12345678 as the positive control. The yeast cells of *AaABCG40* transformant, *AtPDR12* transformant and the control (transformed with the empty vector pDR196) were incubated in half-strength SD medium containing 50 μM ABA, respectively, and the intracellular contents were determined. Yeast-expressing *AaABCG40* exhibited higher ABA content, with 1.7-4.8 folds of that detected in the control at the same time point (**Figure 5**). And the positive control (*AtPDR12* transformant) also accumulated more ABA than that of empty vector control (**Figure 5**). The yeast cells expressing *AaABCG40* showed more efficiency in ABA uptake and took up ABA faster than the control. These results indicate that AaABCG40 was an ABA transporter in yeast.

### Overexpression of AaABCG40 and Its Effects on ABA Regulating the Artemisinin Biosynthesis

In *A. annua*, the artemisinin content was enhanced with ABA treatment through promoting the expression level of the artemisinin biosynthetic genes (Jing et al., 2009). Great progress has been made to reveal the molecular mechanism on ABA regulation of the artemisinin biosynthesis. Previously, AabZIP1 was identified from *A. annua* and proved to activate *ADS* and *CYP71AV1* expressions by binding to their promoters (Zhang et al., 2015). In addition, AaABF3 was reported to positively regulate the artemisinin biosynthesis through directly binding to *ALDH1* promoter (Zhong et al., 2018). To further identify the function of AaABCG40 in *A. annua*, the OE-*AaABCG40-*26, *iAaABCG40*-12 and wild type cutting seedlings were prepared to be treated by exogenous ABA. Subsequently, the transcription level of *CYP71AV1* was measured by RT-qPCR. The results showed that the transcription level of *CYP71AV1* increased rapidly after the ABA treatment and peaked at 6 h, the expression of *CYP71AV1* in wild type increased 1.83-fold (**Figure S5**). The *CYP71AV1* transcription level in OE-*AaABCG40*-26 increased 2.27-fold at 6 h, while the *CYP71AV1* transcription level in i*AaABCG40-12* increased 1.58-fold at 6 h (**Figure S5**), suggesting that *CYP71AV1* in OE-*AaABCG40* plants showed more sensitive to exogenously ABA than that in both wild-type and *iAaABCG40* plants. Taken together, AaABCG40 might be involved in ABA transport in *A. annua*.

### *AaABCG40*-Overexpression Plants Showed More Tolerant to Drought in *A. annua*

**Figure 6:**
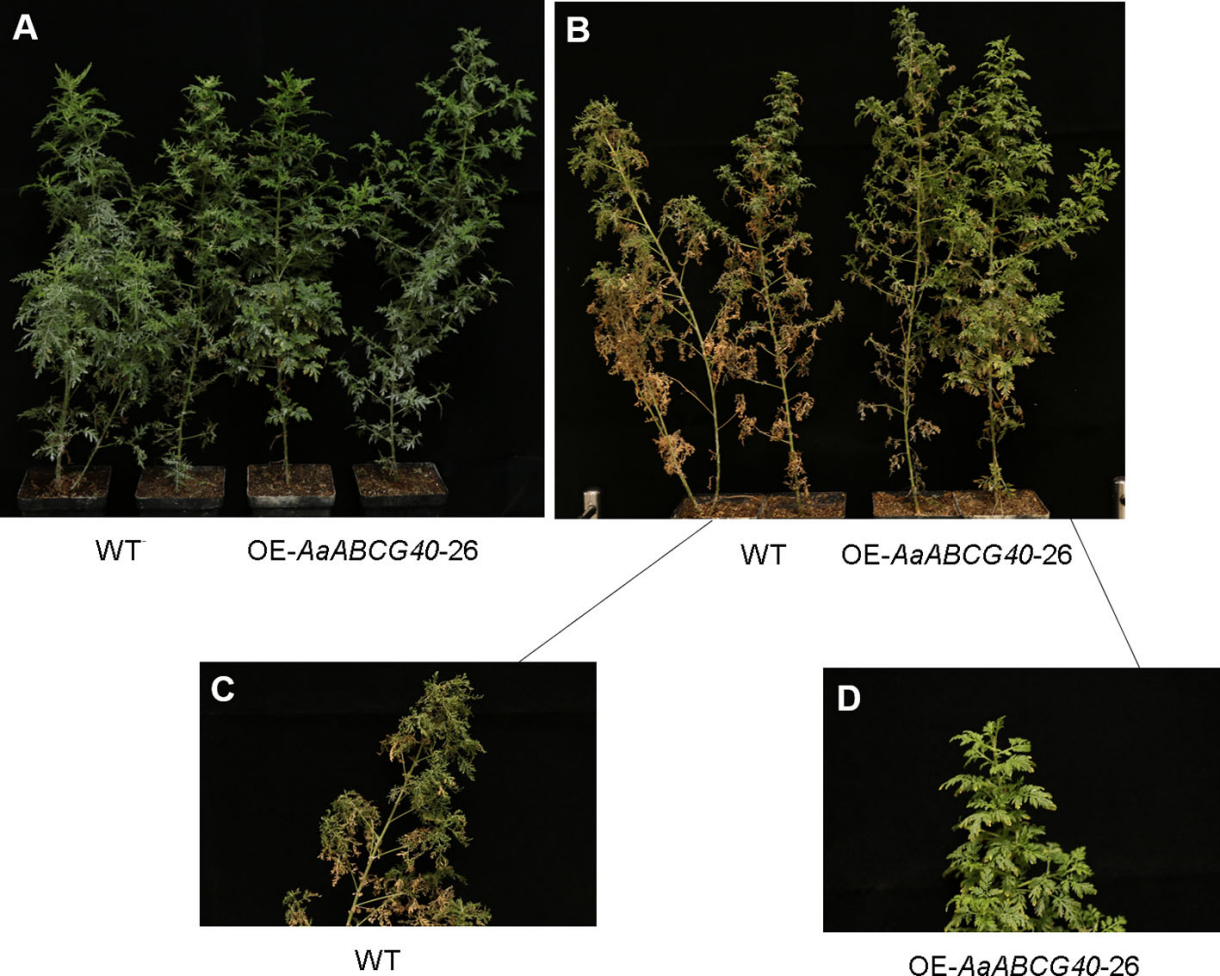
*AaABCG40*-overexpression transgenic *A. annua* showed better tolerance under drought stress. **(A)** Two-month-old cutting seedlings of OE- *AaABCG40* transgenic plants and wild-type *A. annua* plants were cultivated in pots and watered well in the growth chamber under a 16-h light/8-h dark cycle at 25°C for a week. **(B)** Water was withheld for 14 days. **(C)** Wild-type *A. annua* plant was cultivated after water supply was absolutely stopped for 14 days. **(D)** OE-*AaABCG40* transgenic plant was cultivated after water supply was absolutely stopped for 14 days.

The phytohormone ABA participates in many physiological processes, such as photosynthesis, abiotic stress, seed germination and stomatal regulation (Savouré et al., 1997; Zhou et al., 2006; Cutler et al., 2010; Kim et al., 2012). In particular, drought improves ABA biosynthesis and results in the closure of stomata in plants (Zhang et al., 2001; Shinozaki and Yamaguchi-shinozaki, 2007). As described above, AaABCG40 functioned as ABA importer might be involved in drought-stress response in *A. annua*. We prepared the OE-*AaABCG40-*26, *iAaABCG40*-12 and wild type cutting seedlings to test the ability of tolerance to drought. We found that leaves of the OE-*AaABCG40* plant wilted more slowly than those of the control under drought stress (**Figure 6**). And *iAaABCG40*-12 transgenic plants exhibited more rapid wilting than those of the control (**Figure S6**). Taken together, these results indicated that overexpression of *AaABCG40* significantly improved drought tolerance in *A. annua*.

## Discussion

### AaABCG40 Was Involved in ABA Transport

ABA plays an important role in responses to environmental changes, such as drought stress, the regulation of stomatal guard-cell and seed germination. In plants, ABA is predominantly synthesized in vascular tissues, and delivered to the stomatal guard-cell (Hartung et al., 2002; Koiwai et al., 2004; Weathers et al., 2006; Endo et al., 2008). Many molecules involved in ABA transport have been identified. In *Arabidopsis*, pleiotropic drug resistance transporter PDR12 (AtPDR12)/AtABCG40 was reported to act as ABA importer (Kang et al., 2010). AtPDR12 was mainly expressed in the young leaves, and also in primary and lateral roots. When *AtABCG40* was expressed in both YMM12 yeast and tobacco BY2 cells, the results indicated that AtABCG40 functioned as ABA transporter. Besides, *atabcg40* mutants wilted faster than those of control and exhibited a strongly delayed response to ABA.

Here, we characterized a PDR transporter AaPDR4/AaABCG40 from *A. annua*. RT-qPCR showed that *AaABCG40* was mainly expressed in trichomes, young leaves and roots (**Figure 1D**). Notably, the GUS staining also exhibited that AaABCG40 was active in the vascular tissues of leaves, trichomes, stems, and roots (**Figure 2**). Interestingly, ABA is predominantly produced in the vascular tissues (Cheng et al., 2002; Koiwai et al., 2004; Endo et al., 2008). If AaABCG40 acted as a carrier for the delivery of ABA into cells, it would be localized to plasma membrane in plants. AaABCG40 fused GFP protein was localized to plasma membrane with the marker protein in tobacco (**Figure 3**), indicating that AaABCG40 had the ability to transport ABA into the cells. In conclusion, we hypothesis that AaABCG40 located at the plasma membrane is important factor in the ABA transport. A heterologous yeast expression system is a useful method for identifying the function of transporters (Morita et al., 2009; Yu and De Luca, 2013; Fu et al., 2017). To assess whether AaABCG40 functions as an ABA transporter or not, *AaABCG40* cDNA was expressed in the yeast strain AD12345678. The results showed that yeast expressing AaABCG40 consistently accumulated more ABA than controls containing the empty vector along the same time course (**Figure 5**). In addition, when OE-*AaABCG40-*26, *iAaABCG40*-12 and wild type cutting seedlings were treated by exogenous ABA, OE-*AaABCG40* plant showed more sensitive to exogenously ABA (**Figure 4**). Besides, we analyzed ABA content in the transgenic lines using an ABA ELISA kit. The results revealed that leaves of *AaABCG40*-overexpression transgenic *A. annua* plants contained a higher level of ABA than wild type (**Figure S7**). On the contrary, ABA content in leaves of *AaABCG40-*RNAi transgenic *A. annua* plants was reduced, compared with wild type (**Figure S7**).

In our investigation, these data preferentially suggest that AaABCG40 would be involved in ABA transport based on four findings: *i*) the amino acid sequence of AaABCG40 belonging to the full-length size PDR subfamily, contains two NBDs (nucleotide-binding domains) and two TMDs (transmembrane domains) (**Figure 1B**), *ii*) AaABCG40 is localized to plasma membrane and active in trichomes, the vascular tissues of leaves and roots, where ABA is mainly biosynthesized (**Figures 2** and **3**), *iii*) when *AaABCG40* was transferred into yeast AD1-8, yeast expressing *AaABCG40* could accumulate ABA faster than controls containing the empty vector (**Figure 5**), *iiii*) the *AaABCG40-*overexpression transgenic plant showed a higher expression of *CYP71AV1* with the exogenous ABA treatment (**Figure S5**). Taken together, these results indicated that AaABCG40 was involved in ABA transport.

### Effects of AaABCG40 on ABA Regulating the Artemisinin Biosynthesis

To identify the function of AaABCG40, we generated *AaABCG40*-RNAi and *AaABCG40*-overexpression transgenic *A. annua* plants. The artemisinin contents of the leaves in *AaABCG40* overexpressing and *AaABCG40* RNAi transgenic lines measured by HPLC were significantly higher and lower, respectively, than that of wild type plants (**Figures 4B, E**). As we know, exogenous ABA treatment enhances artemisinin accumulation in *A. annua* (Jing et al., 2009). Overexpression of an ABA receptor gene *AaPYL9* also observably enhanced the artemisinin production in *A. annua* (Zhang et al., 2013). Besides, AabZIP1 and AaABF3 involved in ABA signaling were reported to positively regulate the artemisinin biosynthesis. Overexpression of *AabZIP1* and *AaABF3* respectively increased the artemisinin contents, while reducing the expression of *AabZIP1* and *AaABF3* respectively resulted in a decrease in artemisinin contents (Zhang et al., 2015; Zhong et al., 2018). We analyzed the expression level of *AabZIP1* and *AaABF3* in *AaABCG40*-RNAi and *AaABCG40*-overexpression transgenic *A. annua* plants. The results showed that the expression of both *AaZIP1* and *AaABF3* were reduced in *AaABCG40* RNAi lines, while overexpressing *AaABCG40* significantly increased the transcript levels of *AaZIP1* and *AaABF3* in *AaABCG40*-overexpression transgenic lines (**Figure S8**). RT-qPCR analysis also showed that the expressions of *ADS*, *CYP71AV1*, *DBR2,* and *ALDH1* were increased in *AaABCG40*-overexpression transgenic lines (**Figure 4C**). And we also noticed that the transcript level of *ADS* and *ALDH1* were not downregulated in *AaABCG40*-RNAi transgenic lines (**Figure 4F**). In plants, several ABA transporters are synergistically responsible for ABA transport. ABA content in leaves of *AaABCG40-*RNAi transgenic *A. annua* plants was slightly lower than that in the wild type plants. Moreover, the artemisinin biosynthesis has the very complex regulatory network. Previous research indicated that both biotic factors and abiotic factors observably influence the artemisinin biosynthesis in *A. annua*. According to these results, we speculated that overexpressing *AaABCG40* increased ABA accumulation, which activated the expression of the transcription factor genes in ABA signaling pathway to promote the artemisinin biosynthesis in *AaABCG40*-overexpression transgenic lines.

### AaABCG40 Modulates Drought Tolerance

ABA is rapidly accumulated when plants are exposed to drought stress (Leung and Giraudat, 1998). If AaABCG40 functioned as ABA importer, AaABCG40 would be involved in drought-stress response in plants. We detected the ability of drought resistance using OE-*AaABCG40-*26, *iAaABCG40-*12, and wild type cutting seedlings. As expected, the leaves of OE-*AaABCG40-*26 seedlings wilted more slowly than those of wild type (**Figure 6**). In addition, the next generation of *iAaABCG40-12* and OE-*AaABCG40-*26 transgenic plants were analyzed the ability of drought resistance and the water loss. The seeds of *iAaABCG40-12,* OE-*AaABCG40-26* transgenic plant and wild-type *A. annua* plants were cultivated in pots and watered well in the growth chamber under a 16-h light/8-h dark cycle at 25°C for 1 month. Then the water supply was absolutely stopped. For drought treatment, water was withheld for a period of 20 days. As **Figure S9** shown, the seedlings of OE-*AaABCG40-*26 transgenic plants wilted more slowly and also lost water more slowly than wild type and *iAaABCG40-12* seedlings. These results suggested that the leaves of OE- *AaABCG40-*26 seedlings accumulated more ABA than those of wild type, and repression the expression of *AaABCG40* impaired the ability of rapid response to drought stress.

## Data Availability Statement

All datasets generated for this study are included in the article/**Supplementary Material**. Accession Numbers: AaABCG40 (KR559559.1), AaPDR3 (KR153482), AtPDR12 (NM_001332173.1), AtPDR13 (NM_001341001.1), NpPDR1 (Q949G3.1), NtPDR1 (Q76CU2.1), SpTUR2 (O24367.1).

## Author Contributions

XF and KT designed the research. XF and HL performed the experiments. XF, DH, BP, XY, and YW carried out vector construct, expression analysis, transgene plant generation, subcellular localization and yeast assay. XF and KT drafted the manuscript. CW, PL, QP, JZ, HQ, and XS revised the manuscript. All authors contributed to the article and approved the submitted version.

## Funding

This research was supported by the National Science Foundation of China (18Z103150043); China Postdoctoral Science Funding (2018M630435).

## Conflict of Interest

The authors declare that the research was conducted in the absence of any commercial or financial relationships that could be construed as a potential conflict of interest.

The reviewer LZ declared a past co-authorship with several of the authors XF, LL, KT to the handling Editor.
