# Supplementary material for: AaABCG40 Enhances Artemisinin Content and Modulates Drought Tolerance in Artemisia annua
Source: Front Plant Sci. 2020 Jun 26;11:950. doi: 10.3389/fpls.2020.00950 (PMC7333515; doi:10.3389/fpls.2020.00950)
Supplement: Supplementary file 1 [file DataSheet_1.pdf]

Supplementary Information

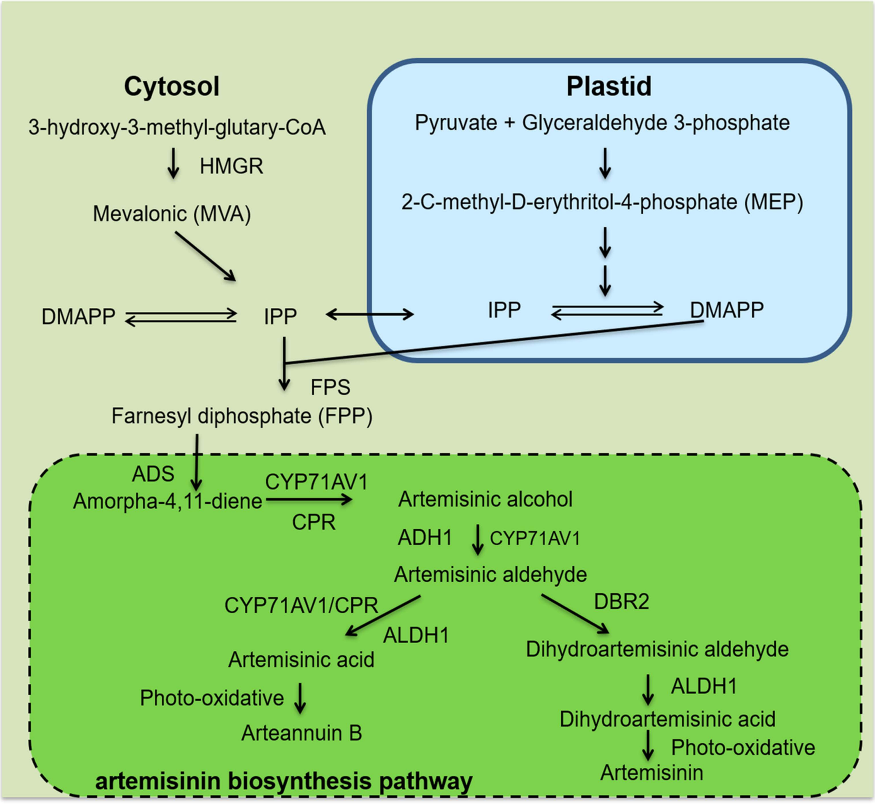

Figure S1. The artemisinin biosynthetic pathway. HMGR, 3-hydroxy-3-methylglutaryl -CoA reductase; DXS, 1-deoxyxylulose 5-phosphate synthase; DXR, 1-deoxyxylulose 5-phosphate reductoisomerase; DMAPP, dimethylallyl diphosphate; IPP, isopentenyl diphosphate; FPS, farnesyl diphosphate synthase; ADS, amorpha-4,11-diene synthase; CYP, cytochrome P450; CYP71AV1, cytochrome P450-dependent hydroxylase; CPR, cytochrome P450 oxidoreductase; DBR2, double bond reductase 2; ALDH1, aldehyde dehydrogenase 1; ADH1, alcohol dehydrogenase; CYB5, cytochrome b5 monooxygenase.

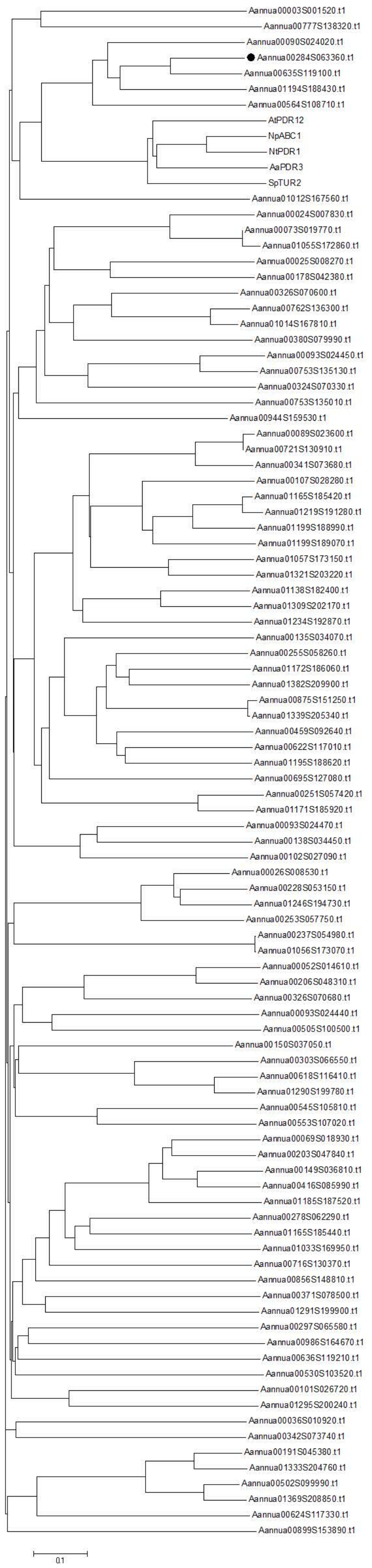

Figure S2. Phylogenetic tree showing the relationship ABC transporters in *A. annua* compared with AtPDR12 from *Arabidopsis*. The tree presented here is a neighbor-joining tree based on amino acid sequence alignment. The candidate gene *AaABCG40* is marked by the black circle.

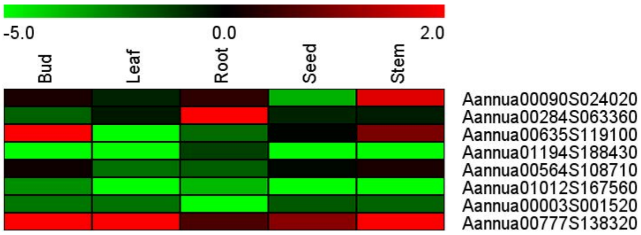

Figure S3. Hierarchical cluster analysis of 8 PDR transporter genes in *A. annua.* The color scale at the top represents the value of log-transformed reads per kilobase per million of mapped reads.

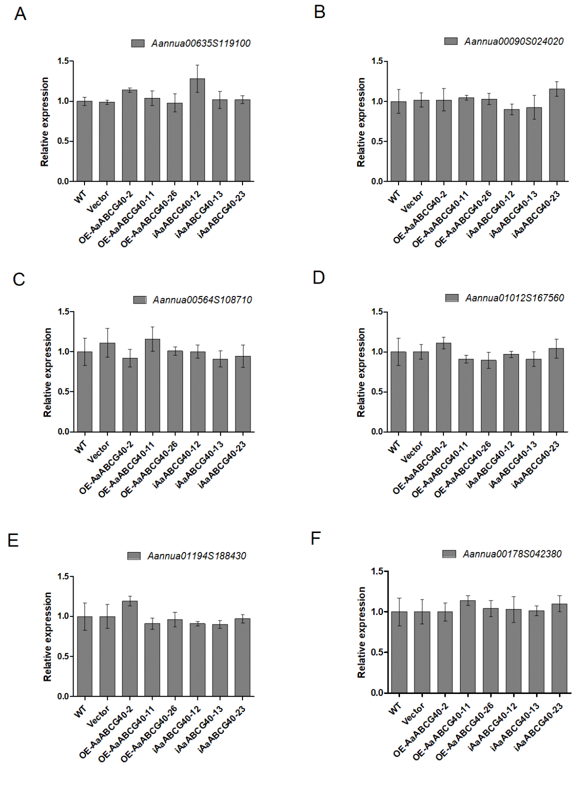

Figure S4. Comparative analyses of *AaABCG* gene expression in wild type (WT), plants transformed with the empty vector (EV), *AaABCG40*-overexpression and *AaABCG40*-RNAi plants. (A) Relative expression of Aannua00635S119100 in WT, EV and *AaABCG40* transgenic *A. annua* lines. **(B)** Relative expression of Aannua00090S024020 in WT, EV and *AaABCG40* transgenic *A. annua* lines. **(C)** Relative expression of Aannua00564S108710 in WT, EV and *AaABCG40* transgenic *A. annua* lines. **(D)** Relative expression of Aannua01012S167560 in WT, EV and *AaABCG40* transgenic *A. annua* lines. **e** Relative expression of Aannua01194S188430 in WT, EV and *AaABCG40* transgenic *A. annua* lines. f Relative expression of Aannua00178S042380 in WT, EV and *AaABCG40* transgenic *A. annua* lines. The error bars represent the means ± SD from three biological replicates.

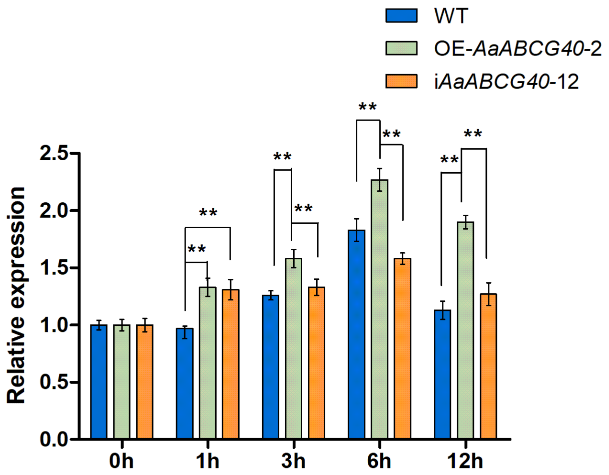

Figure S5. Relative expression of *AaCYP71AV1* in wild type (WT), OE-*AaABCG40*-2 and *AaABCG40*-RNAi-12 plants with the ABA treatment. *ACTIN* was used as internal control. The error bars represent the means ± SD from three replicates. P < 0.0 5, P < 0.01, student's *t*-test.

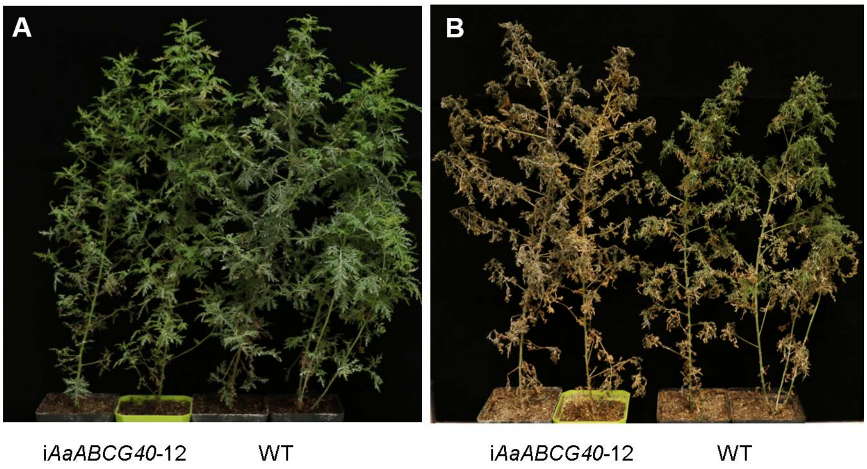

Figure S6. *AaABCG40*-RNAi transgenic *A. annua* showed worse tolerance under drought stress. (A) Two-month-old cutting seedlings of *AaABCG40*-RNAi transgenic plants and wild-type *A. annua* plants were cultivated in pots and watered well in the growth chamber under a 16-h light/8-h dark cycle at 25°C for a week. **(B)** Water was withheld for 14 days.

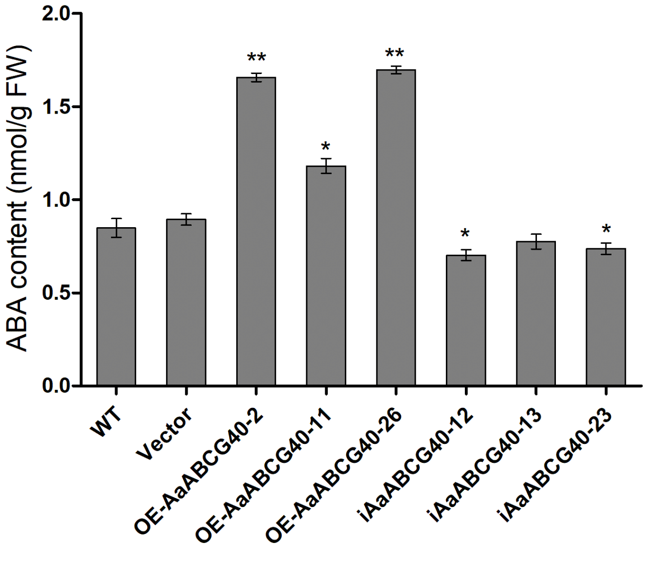

Figure S7. ABA levels measured in the leaves of wild type (WT), OE-*AaABCG40*-2 and *AaABCG40*-RNAi-12 plants. The error bars represent the means ± SD from three replicates. P < 0.0 5, P < 0.01, student's *t*-test.

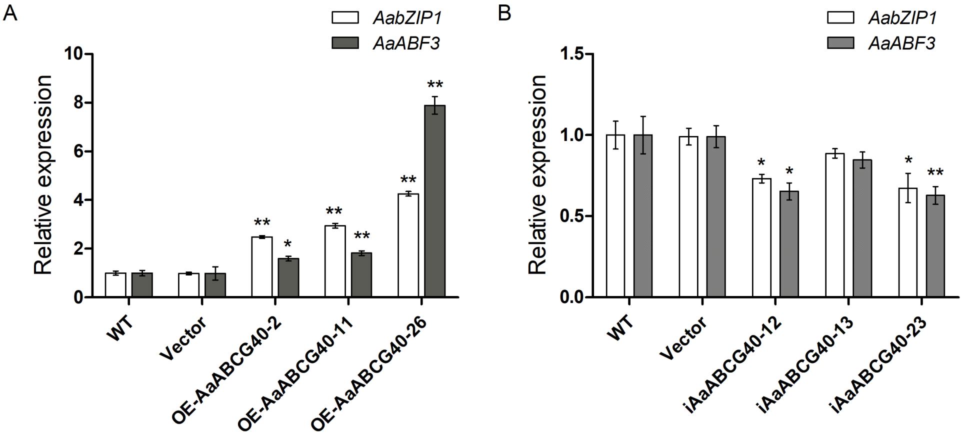

Figure S8. Relative expression of *AaZIP1* and *AaABF3* in wild type (WT), plants transformed with the empty vector (EV), *AaABCG40*-overexpression and *AaABCG40*-RNAi plants. (A) Relative expression of *AaZIP1* and *AaABF3* in WT, EV and *AaABCG40*-overexpression transgenic *A. annua* lines. **(B)** Relative expression of *AaZIP1* and *AaABF3* in WT, EV and *AaABCG40*-RNAi transgenic *A. annua* lines. *ACTIN* was used as internal control. The error bars represent the means ± SD from three technical replicates. P < 0.0 5, P < 0.01, student's *t*-test.

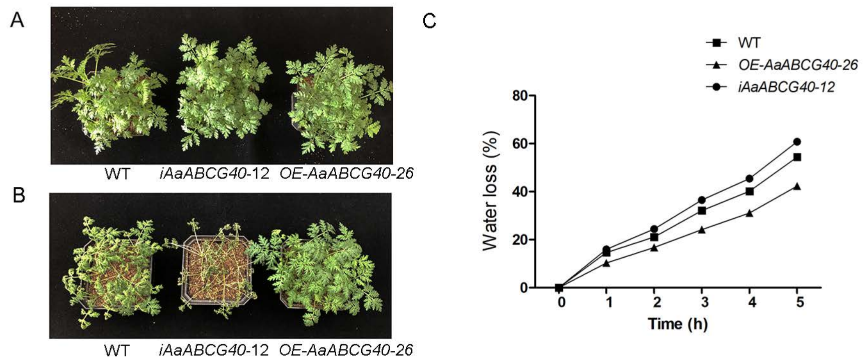

Figure S9. The next generation of *AaABCG40*-overexpression transgenic *A. annua* showed better tolerance under drought stress. (A) The seeds of OE-*AaABCG40-26* transgenic plant and wild-type *A. annua* plants were cultivated in pots and watered well in the growth chamber under a 16-h light/8-h dark cycle at 25°C for one month. (B) Water was withheld for 20 days. (C)Time course of water loss from the leaves of 4-week-old wild-type and OE-*AaABCG40-26 A. annua* plants. Three independent experiments were performed, and three independent plants were used to calculate. The error bars represent the means ± SD from three technical replicates.

Table S1. Primers used in this study.

No.	Primer name	Use	Primer sequence 5´-3´	Restriction enzyme
1	AaABCG40-FP1	cloning	TGTAACAATGGATGGAACTGACTGGAAT	-
2	AaABCG40-RP1	cloning	CGACTGCAATCCAATACCAGTAAGC	
3	AaABCG40-FP	qPCR	GGGAAACAAACGATTTTATCCAGAGC	
4	AaABCG40-RP	qPCR	CCAAGGAAAGTAAGAGCCACAGCATA	
5	β-actin-FP	qPCR	CCAGGCTGTTCAGTCTCTGTAT	
6	β-actin-RP	qPCR	CGCTCGGTAAGGATCTTCATCA	
7	BamHI-AaABCG40-FP	cloning	CTCTCTCTCCAACTTGGATCCATGGAACTGACTGGAATA	*BamH*I
8	AaABCG40-SpeI-RP	cloning	CCCTTGCTCACCATACTAGTTCTCTTCTGGAAATTAAAGGAT	*Spe*I
9	AaABCG40-RNAi-FP	cloning	CACCGGTGAACGAGTTCCTTGGCC	
10	AaABCG40-RNAi-RP	cloning	CATCGAAGGTAATTGAATGTGGTTC	
11	Pro- AaABCG40-FP	cloning	TTGGTGTGTTTGTTTTTGCCTATTTATG	
12	Pro- AaABCG40-RP	cloning	CTTTATTCCAGTCAGTTCCATCCATTG	
13	PstI-ProAaABCG40 -FP	cloning	TGCACTGCAGTTGGTGTGTTTGTTTTTGCCTAT	*Pst*I
14	ProAaABCG40-BamHI-RP	cloning	CGGGATCCTGTTACAAAAGATCAATTGATC	*BamH*I
15	AaADS-F	qPCR	GGACTAGGTTCAGGCTATG	
16	AaADS-R	qPCR	AAGCATGTAATTGACCACC	
17	AaCYP71AV1-F	qPCR	TCATTTCAGTCGCTT	
18	AaCYP71AV1-R	qPCR	CCAGTTTGCCTCAGTA	
19	AaDBR2-F	qPCR	ACTGCTGGTGGCTTTCTTA	
20	AaDBR2-R	qPCR	ACCCTCGACTTGTTCCTTA	
21	AaALDH1-F	qPCR	CAGTTTCTGACCCAAATCCAGGTTGA	
22	AaALDH1-R	qPCR	TCGGAGTAGTTGGTCACAT	
23	PDR196-AaABCG40-FP	cloning	AGTGGATCCCCCGGGCTGCAGATGGATGGAACTGACTGGAATA	
24	PDR196-AaABCG40-RP	cloning	CTTGATATCGAATTCCTGCAGTTATCTCTTCTGGAAATTAAAG	
25	PDR196-AtPDR12-FP	cloning	AGTGGATCCCCCGGGCTGCAGATGGAGGGAACTAGTTTTCACC	
26	PDR196-AtPDR12-RP	cloning	GTACCGGGCCCCCCCTCGAGCTATCGTTTTTGGAAATTGA	
27	Aannua00178S042380-RT-FP	qPCR	CTCAACTCCGTGGTTCCAACTCCT	
28	Aannua00178S042380-RT-RP	qPCR	TATTGAACCAGAAAATGGACGGCT	
29	Aannua00635S119100-RT-FP	qPCR	TCCCGTGAAGAAAATGACGAAGA	

30	Aannua00635S119100-RT-RP	qPCR	ATCTTCATCAGCGGCATTCACAA	
31	Aannua01194S188430-RT-FP	qPCR	AGGAGCACGAAGCAGAAGCCAAA	
32	Aannua01194S188430-RT-RP	qPCR	TGTAAAGACGCCAGTTTCAATGC	
33	Aannua00090S024020-RT-FP	qPCR	AGGACACAAGATAACGACGAGGA	
34	Aannua00090S024020-RT-RP	qPCR	CAACAAGCGTAAACTCGCCTCCAG	
35	Aannua00564S108710-RT-FP	qPCR	GCTTGGGCACAATGGGTGGGTAT	
36	Aannua00564S108710-RT-RP	qPCR	AATAGCCTTGTCTAATGGAGGGAGT	
37	Aannua00178S042380-RT-FP	qPCR	TACTGAATGGGGTAACAGGGAAG	
38	Aannua00178S042380-RT-RP	qPCR	ATTGAACCAGAAAATGGACGGCT	
39	AabZIP1-RT-FP	qPCR	CGATGTTTCCGAAGCCGTAT	
40	AabZIP1-RT-RP	qPCR	TGCTAACCCGTCCGATGA	
41	AaABF3-RT-FP	qPCR	GCTGCTTTCAACTTCAACACTTC	
42	AaABF3-RT-RP	qPCR	ATAAATCACGTTGAGCCATGCT	
